# CAR-T therapy alters synthesis of platelet-activating factor in multiple myeloma patients

**DOI:** 10.1186/s13045-021-01101-6

**Published:** 2021-06-09

**Authors:** Mengying Ke, Liqing Kang, Ling Wang, Shu Yang, Yajun Wang, Haiyan Liu, Chunyan Gu, Hongming Huang, Ye Yang

**Affiliations:** 1 Nanjing Hospital of Chinese Medicine affiliated to Nanjing University of Chinese Medicine, Nanjing, China; 2 School of Medicine and Holistic Integrative Medicine, Nanjing University of Chinese Medicine, 138 Xianlin Road, Nanjing, 210023 China; 3 Shanghai Unicar-Therapy Bio-Medicine Technology Co., Ltd, Shanghai, China; 4 Department of Hematology, The Affiliated Hospital of Nantong University, Nantong, 226001 Jiangsu China

**Keywords:** Platelet-activating factor, Multiple myeloma, CAR-T therapy, Cytokine release syndrome, LPCAT1

## Abstract

The chimera antigen receptor (CAR) T cell therapy is a novel and potential targeted therapy and has achieved satisfactory efficacy in patients with relapsed or refractory multiple myeloma (MM) in recent years. However, cytokine release syndrome (CRS) and clinical efficacy have become the major obstacles which limit the application of CAR-T in clinics. To explore the potential biomarkers in plasma for evaluating CRS and clinical efficacy, we performed metabolomic and lipidomic profiling of plasma samples from 17 relapsed or refractory MM patients received CAR-T therapy. Our study showed that glycerophosphocholine (GPC), an intermediate of platelet-activating factor (PAF)-like molecule, was significantly decreased when the participants underwent CRS, and the remarkable elevation of lysophosphatidylcholines (lysoPCs), which were catalyzed by lysoPC acyltransferase (LPCAT) was a distinct metabolism signature of relapsed or refractory MM patients with prognostic value post-CAR-T therapy. Both GPC and lysoPC are involved in platelet-activating factor (PAF) remodeling pathway. Besides, these findings were validated by LPCAT1 expression, a key factor in the PAF pathway, associated with poor outcome in three MM GEP datasets of MM. In conclusion, CAR-T therapy alters PAF synthesis in MM patients, and targeting PAF remodeling may be a promising strategy to enhance MM CAR-T therapy.

**Supplementary Information:**

The online version contains supplementary material available at 10.1186/s13045-021-01101-6.

**To the Editor,**

Multiple myeloma (MM) is still an incurable hematological malignancy, as most of MM patients eventually relapse or become refractory [1, 2]. Strikingly, the CAR-T cell therapy has achieved satisfactory efficacy in patients with relapsed or refractory MM in recent years [3]. However, cytokine release syndrome (CRS) and clinical efficacy have become the major obstacles which limit the application of CAR-T in clinics [4]. To explore the potential biomarkers in plasma, we uniquely imported metabolomics analytic techniques and examined the profile changes of metabolites and lipids in plasma from 17 relapsed or refractory MM patients post-CAR-T therapy (Fig. 1a). The clinical characteristics of 17 participants are presented in Additional file 1: Table S1.

**Fig. 1:**
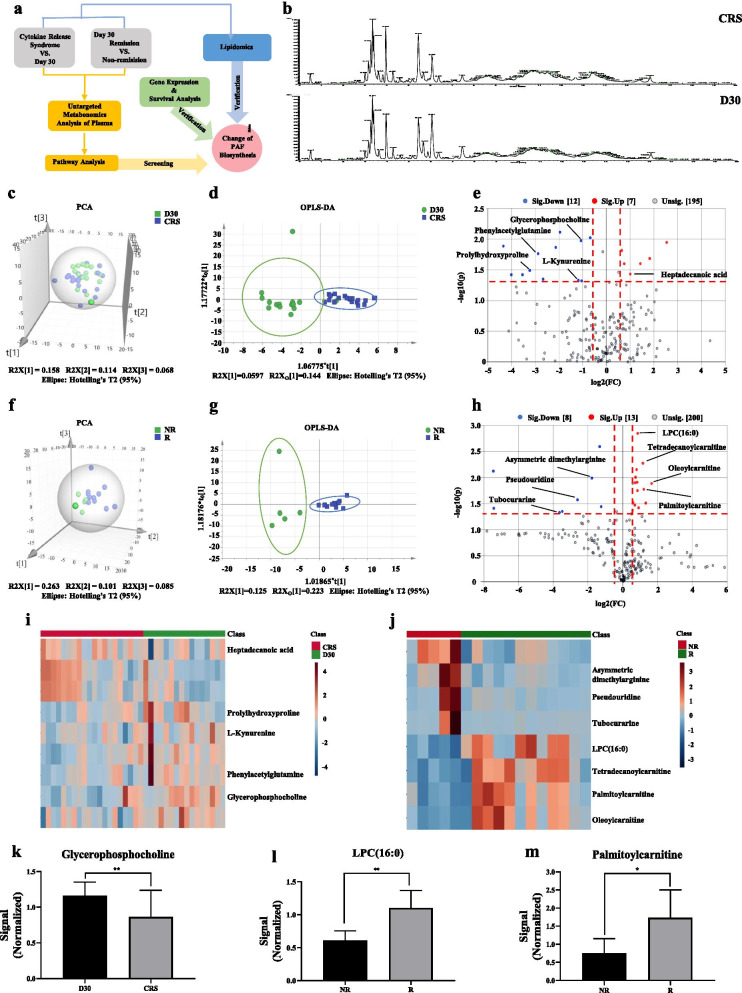
The metabolomic characteristics of the screening set. **a** Study design including untargeted plasma metabolomics, lipidomic and GEP analysis. **b** Typical chromatograms of TIC in plasma samples from CRS group (containing participants with CRS within the first week post-infusion and without CRS at day 30 (D30) post-infusion). **c** Score plot of PCA based on the data of ESI^+^ mode from CRS group. **d** Score plot of OPLS-DA based on the data of ESI^+^ mode from CRS group. **e** Volcano plot based on the data of ESI^+^ mode from CRS group. **f** Score plot of PCA based on the data of ESI^+^ mode from comparative efficacy group (containing participants in remission (R) and non-remission (NR) at day 30 after infusion). **g** Score plot of OPLS-DA based on the data of ESI^+^ mode from comparative efficacy group. **h** Volcano plot based on the data of ESI^+^ mode from comparative efficacy group. **i** Heatmap showed 5 differential metabolites identified from the comparison of CRS vs D30. **j** Heatmap showed 7 differential metabolites identified from the comparison of R vs NR. **k** The changes of GPC level in CRS group. **l**–**m** The changes of LPC(16:0) and palmitoylcarnitine levels in comparative efficacy group. (*p < 0.05, **p < 0.01)

We first detected the differential metabolites in CRS group including participants with CRS within the first week post-infusion and without CRS at day 30 post-infusion (Additional file 1: Fig. S1) and comparative efficacy group (CE group) including participants with remission and non-remission at day 30 post-infusion by UPLC-MS in positive electrospray ionization (ESI^+^) mode (Additional file 2: Table S2, S3). The representative total ion chromatograms (TIC) for all participants demonstrated the well separation of metabolites in plasma (Fig. 1b). Subsequently, the principal component analysis (PCA) and the orthogonal partial least squares discriminant analysis (OPLS-DA) (Additional file 2: Fig. S2)showed significant differences among samples from CRS group (Fig. 1c, d) or CE group (Fig. 1f, g). By measuring the variable importance, 19 and 21 substances were distinguished in CRS group (Fig. 1e) and CE group (Fig. 1h), respectively. After matching KEGG modules, 5 and 7 differential metabolites were identified from the two comparisons, respectively, shown as a heatmap (Fig. 1i, j). Oxidized glycerophosphocholine (Ox-GPC) is PAF-like molecule combining with PAF receptor [5]. Platelet-activating factor (PAF), known as 1-alkyl-2-acetyl-sn-glycero-3-phosphocholine [6], is produced through remodeling or de novo pathway involved in different biological pathways of inflammation [7], tumor cell growth [8], and invasion [9, 10]. Compared to the patients without CRS at day 30, the plasma level of GPC was significantly decreased in patients with CRS (Fig. 1k), indicating that the activation of GPC oxidative phosphorylation and the intensification of PAF synthesis were associated with CRS. Intriguingly, Lysophosphatidylcholine (LPC), as an intermediate in remodeling pathway to form PAF with the addition of an acetyl group, is catalyzed by LPC acyltransferase (LPCAT) [11]. The plasma levels of LPC(16:0) and palmitoylcarnitine were significantly increased in relieved participants from CE group (Fig. 1l, m).

We further conducted targeted metabolomics to explore LPCs volume, and as shown in Fig. 2a, disorderedly metabolic pathways were involved in PAF synthesis [12]. Moreover, the area under receiver operating characteristic (ROC) curve of LPC(16:0) was 0.9833, suggesting that LPC could serve as a potential plasma biomarker for prognosis of relapsed or refractory MM (Fig. 2b). Then, to validate reliable plasma biomarkers for prognosis of CAR-T therapy (Additional file 3: Table S4), we detected TIC, PCA, and OPLS-DA (Additional file 3: Fig. S3) of lipidomic profiling in CE group represented in Fig. 2c–e, respectively. LPC(O-24:0), LPC(20:4) and LPC(16:1/0:0), significantly heightened in relieved participants at day 30 post-CAR-T therapy among 44 differential lipids (Fig. 2f–i). Heatmap showed marked elevation of the three LPCs compared with top 20 differential lipids (Fig. 2j). In addition, LPCAT1, one of LPCAT family members, is crucial for lipid droplet remodeling and LPC metabolism [11]. Therefore, we assessed the gene expression of LPCAT1 in normal people (NP), MGUS and MM bone marrow plasma cells based on GEP dataset. The LPCAT1 mRNA was remarkably increased in MM patients compared with MGUS and NP (*p* = 0.0332; Fig. 2k). Furthermore, unlike other members in LPCAT family (Additional file 4: Fig. S4), higher LPCAT1 expression was associated with poor overall survival (OS) in MM patients (TT2, GSE2658) (*p* = 0.0378; Fig. 2l). This finding was also verified in other two independent cohorts, HOVON-65 clinical trial (*p* = 0.0052; Fig. 2m) and the APEX phase III clinical trial (*p* = 0.0003; Fig. 2n). Additionally, we found that LPCAT1 mRNA was significantly increased in relapsed MM patients compared with newly diagnosed patients from TT2 cohort (GSE38627) (*p* = 0.0065; Fig. 2o).

**Fig. 2:**
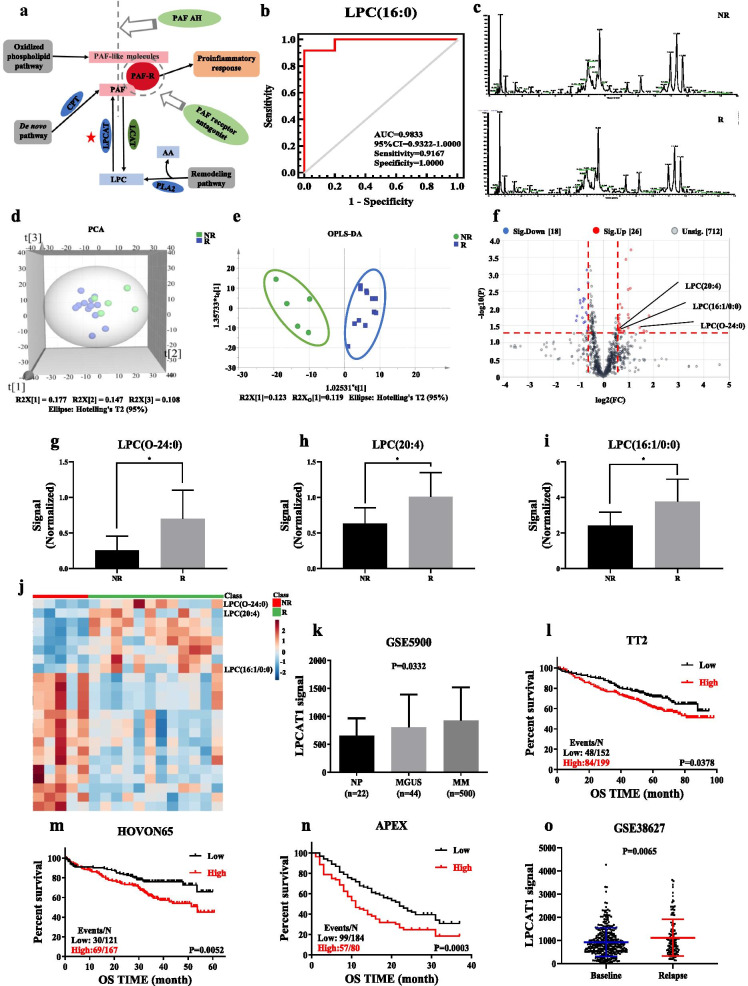
The lipids characteristics of the validation set. **a** PAF synthesis pathway. **b** ROC analysis for LPC(16:0) (AUC: 0.9833, 95% confidence interval: 0.9322–1.0000). **c** Typical chromatograms of TIC in plasma samples from comparative efficacy group. **d** Score plot of PCA based on the data of ESI^+^ mode from comparative efficacy group. **e** Score plot of OPLS-DA based on the data of ESI^+^ mode from comparative efficacy group. **f** Volcano plot based on the data of ESI^+^ mode from comparative efficacy group. **g**–**i** Changes of LPC(O-24:0), LPC(20:4) and LPC(16:1/0:0) contents in plasma of comparative efficacy group. **j** Heatmap showed that LPC(O-24:0), LPC(20:4) and LPC(16:1/0:0) were significantly increased that were identified from the comparison of R vs NR. **k** The mRNA level of LPCAT1 in bone marrow plasma cells from NP (*n* = 22), MGUS (*n* = 44) and MM (*n* = 500). LPCAT1 expression was significantly increased in MM samples by ordinary one-way ANOVA test. **l**–**n** High LPCAT1 expression in MM patients was correlated with poor OS in TT2 cohort, HOVON-65 clinical trial and APEX phase III clinical trial by log-rank test. **o** The expression of LPCAT1 mRNA at baseline (previously published under GSE2685, *n* = 351) and relapse patients (*n* = 130). LPCAT1 expression was markedly increased in the relapsed MM patients by log-rank test. (*p < 0.05, **p < 0.01)

Our study demonstrated that CAR-T therapy could inhibit the remodeling pathway of PAF synthesis via inactivating LPCAT1 and lead to the elevation of plasma LPCs. We also propose that LPCAT1 may act as an oncogene in MM, which is available to be targeted in clinics particularly in CAR-T therapy. Collectively, targeting PAF remodeling may be a promising strategy to enhance MM CAR-T therapy.

## Supplementary Information

**Additional file 1**. The characteristics of patients and plasma samples.

**Additional file 2**. Untargeted metabolomics.

**Additional file 3**. Lipidomics.

**Additional file 4**. GEP analysis of other 3 members in LPCAT family including LPCAT2, LPCAT3 and LPCAT4.

## Data Availability

All supporting data are included in the manuscript and supplemental files. Additional data are available upon reasonable request to the corresponding author.

## Abbreviations

CAR: Chimera antigen receptor
MM: Multiple myeloma
CRS: Cytokine release syndrome
GPC: Glycerophosphocholine
PAF: Platelet-activating factor
LPC: Lysophosphatidylcholine
LPCAT: Lysophosphatidylcholine acyltransferase
GEP: Gene expression profiling
R: Remission
NR: Non-remission
ESI: Electrospray ionization
TIC: Total ion chromatograms
PCA: Principal component analysis
OPLS-DA: Orthogonal partial least squares discriminant analysis
VIP: Variable importance in the projection
FC: Fold change
KEGG: Kyoto encyclopedia of genes and genomes
ROC: Receiver operating characteristic
NP: Normal people
MGUS: Monoclonal gammopathy of undetermined significance
OS: Overall survival
