# Supplementary material for: CAR-T therapy alters synthesis of platelet-activating factor in multiple myeloma patients
Source: J Hematol Oncol. 2021 Jun 9;14:90. doi: 10.1186/s13045-021-01101-6 (PMC8191024; doi:10.1186/s13045-021-01101-6)
Supplement: Supplementary file 1 — Additional file 1. The characteristics of patients and plasma samples. [file 13045_2021_1101_MOESM1_ESM.docx]

**Additional file 1:** The characteristics of patients and plasma samples

17 relapsed or refractory MM patients were from a combination of humanised anti-CD19 and anti-BCMA CAR T cells in patients with relapsed or refractory MM: an open-label, single-center, single-arm trial. All participants provided written informed consent in accordance with the ethics committee of the Afﬁliated Hospital of Nantong University in agreement with the Declaration of Helsinki. The characteristics and clinical responses of 17 participants were presented in Additional file 1: Table S1. The plasma samples were collected for separation of plasma at day 1, 2, 3, 4, 5, 6, 7 and 30 of CAR T cell infusion therapy. All samples were stored in a digital-alarm-controlled freezer at -80°C before analysis. IL-6 concentration (in pg/mL) was measured by Cytometric Bead Array（CBA）Kit (Becton, Dickinson and Company, USA) and flow cytometry(Guava easyCyte 6-2L, Merck, USA). IL-6 concentration of >2.2 pg/mL was deemed as indicator of CRS in Fig.S1.

The screening set consisted of CRS group and comparative efficacy group. CRS group included 20 plasma samples from MM patients with CRS and 16 plasma samples from patients without CRS at day30 (D30) after CAR T cell infusion. Comparative efficacy group of included 12 plasma samples from patients in remission (R) and 5 plasma samples from patients in non-remission (NR) at day 30 after CAR T cell infusion.


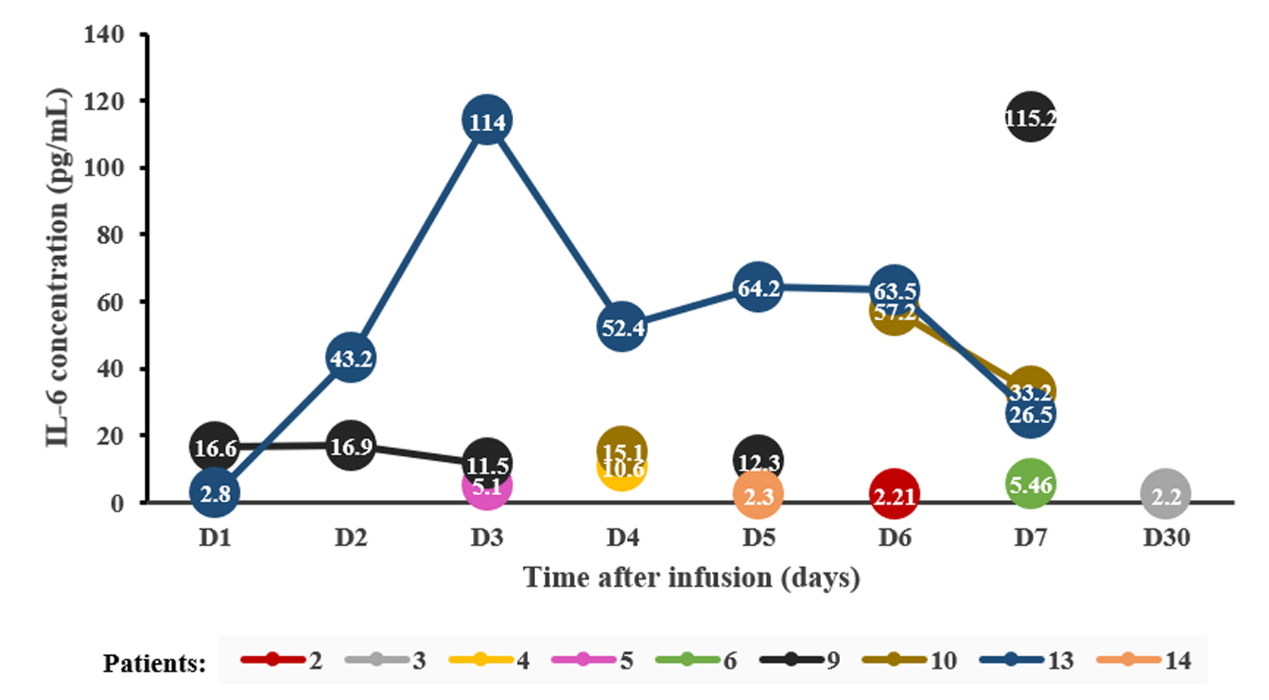


**Fig. S1** Abnormally elevation of serum IL-6 concentrations in participants with CRS.

**Table S1**. Patient characteristics and clinical responses of 17 participants.

| **Patient No.** | **Sex/age** | **ISS stage** | **Durie-Salmon stage** | **Type of myeloma** | **Time from initial MM diagnosis, months** | **Prior lines of therapy** | **Autologous stem cell transplantation** | **Day 0** | | | | | | | | **CAR-T therapy** | | | |
| --- | --- | --- | --- | --- | --- | --- | --- | --- | --- | --- | --- | --- | --- | --- | --- | --- | --- | --- | --- |
|  |  |  |  |  |  |  |  | **Serum M protein, g/L** | **β2-MG, mg/L** | **LDH, IU/L** | **Serum creatinine, mg/Dl** | **Platelets,×10^3^/μL** | **Hemoglobin, g/dL** | **Bone lesions** | **CAR-T infused,×10^6^/kg** | | **Infusion time** | **Peak value of CAR-T, copy number/μg DNA** | **Response at day30^*^** |
| 1 | M/65 | Ⅰ | Ⅲ A | IgD,λ | 52 | 3 | No | ＜0.42 | 2.91 | 178 | 66 | 166 | 127 | No | 10 | | 2019-01-02 | 10047 | VGPR |
| 2 | M/47 | III | Ⅲ A | IgA,λ | 57 | 3 | Yes | 35.9 | 5.53 | 184 | / | 156 | 84 | Yes | 10 | | 2019-01-09 | 3554 | PD |
| 3 | F/59 | I | IA | IgG | 68 | 4 | Yes | ＜0.42 | 1.79 | 150 | / | 136 | 149 | No | 10 | | 2019-07-13 | 51525 | VGPR |
| 4 | F/52 | Ⅲ | Ⅲ A | IgD,λ | 69 | 6 | No | 30.7 | 11.7 | 146 | 82 | 63 | 78 | No | 10 | | 2019-07-25 | 51233 | sCR |
| 5 | F/48 | Ⅲ | Ⅲ | IgG,λ | 38 | 4 | No | 58.5 | 4.93 | 193 | 83 | 139 | 93 | No | 10 | | 2019-09-03 | 12878 | SD |
| 6 | M/44 | / | Ⅱ | IgA,λ | 62 | 4 | No | ＜0.42 | 4.73 | / | 88 | 80 | 111 | No | 10 | | 2019-11-27 | 222598 | SD |
| 7 | M/60 | Ⅱ | ⅡA | IgG,λ | 33 | 2 | Yes | / | 2.3 | 120 | 46 | 167 | 146 | No | 9 | | 2019-03-04 | 33000 | VGPR |
| 8 | M/53 | Ⅱ | Ⅲ A | IgG,κ | 17 | 2 | No | 0.13 | 1.65 | 222.7 | 55.9 | 126 | 135 | No | 2 | | 2020-02-25 | 163561 | CR |
| 9 | F/64 | / | Ⅲ B | κ | 38 | 2 | Yes | / | / | 171 | 61.35 | 60 | 79 | No | 1 | | 2020-06-15 | 104574 | CR |
| 10 | F/66 | Ⅱ | Ⅲ A | IgA,κ | 34 | 3 | No | 0.12 | / | 147.6 | 63 | 41 | 101 | No | 2 | | 2019-07-12 | 35700 | CR |
| 11 | M/49 | / | / | / | 117 | 3 | No | 33.6 | 3.58 | 542 | 71.4 | 131 | 123 | No | 2 | | 2019-08-20 | 21829 | CR |
| 12 | M/54 | Ⅱ | Ⅲ A | IgG,κ | 72 | 1 | Yes | / | 64.27 | 769 | 59.42 | 131 | 68 | Yes | 5 | | 2018-04-24 | 38933 | SD |
| 13 | M/54 | Ⅱ | Ⅲ A | IgA,λ;IgG,κ | 86 | 3 | No | / | 1.74 | 158.1 | 58.6 | 93 | 109 | No | 5 | | 2018-05-09 | 291400 | PR |
| 14 | M/60 | III | IIIA | IgG,λ | 58 | 3 | Yes | 5.54 | 3 | 14 | 48 | 20 | 70 | Yes | 10 | | 2018-04-24 | 1360 | PR |
| 15 | F/65 | I | IA | κ | 26 | 5 | No | / | 17.3 | 175 | 110 | 62 | 70 | Yes | 5.2 | | 2018-09-26 | 488000 | SD |
| 16 | M/64 | Ⅲ | Ⅲ B | IgG,λ | 43 | 2 | No | 54.9 | 14.4 | 286 | 243 | 104 | 77 | Yes | 10 | | 2019-10-21 | 84700 | CR |
| 17 | F/55 | Ⅱ | ⅡA | IgG,κ | 32 | 2 | No | 29.3 | 4.1 | 198 | 52 | 176 | 115 | Yes | 7 | | 2019-07-30 | 14600 | PR |

*sCR: stringent complete response; CR: complete response; VGPR: very good partial response; PR: partial response; SD: stable disease; PD: progression of disease. In this study, sCR, CR, VGPR, PR were divided into remission group and SD, PD were divided into non-remission group.
