# Supplementary material for: CAR-T therapy alters synthesis of platelet-activating factor in multiple myeloma patients
Source: J Hematol Oncol. 2021 Jun 9;14:90. doi: 10.1186/s13045-021-01101-6 (PMC8191024; doi:10.1186/s13045-021-01101-6)
Supplement: Supplementary file 2 — Additional file 2. Untargeted metabolomics. [file 13045_2021_1101_MOESM2_ESM.docx]

**Additional file 2:** Untargeted metabolomics

Sample preparation

Frozen patient plasma was thawed at 4°C and mixed by vortexing. An aliquot of 45 μL of plasma was transferred into a 1.5 mL eppendorf (EP) tube. Then, 135 μL acetonitrile (Merck, USA) containing internal standards (IS) (2-Chloro-L-Phenylalanine (plasma sample-acetonitrile: IS=2000:1)) (Shanghai Yuanye Bio-Technology Co., China) was added to each plasma aliquot. Samples were thoroughly mixed on a vortex mixer for 30 s, incubated at 4°C for 12 h, and centrifuged at 13,000 rpm for 10 min at 4°C. An aliquot of 135 μL of the supernatant was transferred through micromembrane filtration (0.22μm) into a 1.5 mL EP tube. Samples were dried in the SpeedVac sample concentrator (SPD1010-230, Thermo Fisher, USA) at 45°C for 2 h, reconstituted in 120 μL of 50% acetonitrile, vortexed for 15 s, and centrifuged at 13,000 rpm for 10 min at 4°C. An aliquot of 80 μL was transferred into 250 μL glass inserts. These samples were either placed in an autosampler at 4°C for direct analysis or stored at -80°C. Frozen samples were thawed at room temperature and centrifuged at 13,000 rpm for 10 min at room temperature before analysis. Quality control (QC) samples of plasma were also prepared according to the above protocol to monitor retention time and elution order of metabolites.

UHPLC-MS analysis

The Thermo Scientific™ Vanquish™ Horizon UHPLC system coupled to an Thermo Scientific™ Q Exactive hybrid quadrupole-Orbitrap mass spectrometer and operated in full scan mode was used for untargeted analysis of plasma samples. Each sample was run in duplicate in positive ionization mode. An aliquot of 3 μL extracted plasma sample was injected onto an C18 (2.1 × 100 mm, 1.7 μm) column (Fortis Technologies, UK) operating at 40°C. The auto-sampler was conditioned at 4°C. Chromatographic separations were performed by applying a binary mobile phase system. For analyses carried out in the positive ESI mode, mobile phase A consisted of water containing 0.1% (vol/vol) formic acid (Merck, USA) and mobile phase B was acetonitrile. The gradient profile was as follows: 3%B at 0-0.5 min, 20%B at 0.5-1.5 min, 65%B at 1.5-4 min, 95%B at 4-15 min, 3%B at 15-20 min. The flow rate was 0.4 mL/min.

Q Exactive hybrid quadrupole-Orbitrap mass spectrometer was operated in positive ion mode with the ionization temperature of 550°C, an ion-source gas 1 pressure of 50 psi, an ion-source gas 2 pressure of 60 psi, a curtain gas pressure of 35 psi, and an ion-source voltage of 5500 V. The mass spectrometer was operated in the extended dynamic range mode. Mass spectral data were acquired in the profile mode using a scan range of 60–1000 m/z.

QC was assured by randomization of sequence and injection of QC samples between every 10 actual samples.

Data processing and statistics

Raw data files from UHPLC-MS were converted to Analysis Base File (ABF) by Abf Converter and imported into MS-DIAL (version 1.92) software to match the peaks and the metabolites identified by MoNA mass spectral database. The peak height intensity was normalized by QC peak intensity. Then the data set of normalized peak height intensity, retention time (RT), metabolites names and sample numbers were analyzed by SIMCA 14.1 software. SIMCA 14.1 performed a multivariate statistical analysis of the PCA and OPLS-DA and permutations (Fig.S2). The metabolites with *P* value < 0.05 and VIP of >1.0 were considered as statistically significant metabolites. Finally, the significant metabolites with FC >1.5 or <0.67 were deemed as potential biomarkers. Volcano plot, cluster analysis and metabolic pathway analysis were carried out on the website visualization tools of MetaboAnalyst 5.0. Only well-annotated the Human Metabolome Database (HMDB) compounds were selected and correct matching Kyoto Encyclopedia of Genes and Genomes (KEGG) (Table S2, 3).


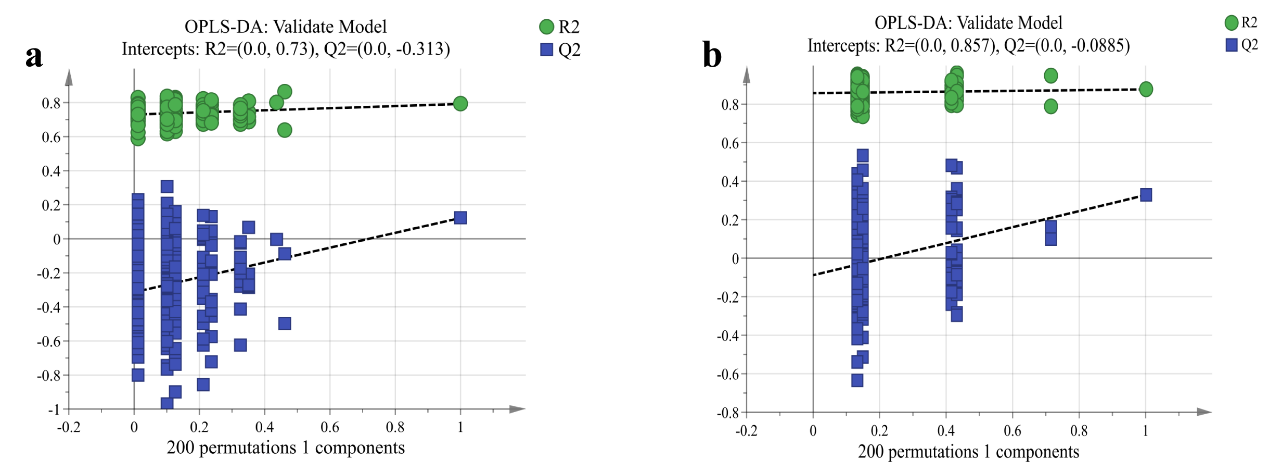


**Fig. S2** **a** Validation of OPLS-DA model in CRS group by 200 permutation tests. R2 = 0.73, Q2 = -0.313. **b** Validation of OPLS-DA model in comparative efficacy group by 200 permutation tests. R2 = 0.857, Q2 = -0.0885.

**Table S2** Differential metabolites between plasma of patients with CRS and without CRS

| **Name** | **m/z** | **Formula** | ***P-value*** | **FC** | **VIP** | **KEGG ID** |
| --- | --- | --- | --- | --- | --- | --- |
| Voriconazole | 350.12192 | C_16_H_14_F_3_N_5_O | 0.00769 | 3.50980 | 1.79482 | C07622 |
| NCGC00347704-02_C24H32O7_2H-Oxireno[1,10a]phenanthro[3,2-b]furan-10(11bH)-one, 5,7-bis(acetyloxy)-3,3a,4,5,6,7,7a,7b,8,8a-decahydro-4,4,7a,11-tetramethyl-, (1aS,3aR,5S,7S,7aR,7bS,8aR,11bR)- | 415.21259 | C_24_H_32_O_7_ | 0.00951 | 4.28700 | 1.22524 | NA |
| Glycerophosphocholine | 258.10974 | C_8_H_21_NO_6_P | 0.01061 | 0.47488 | 1.78119 | C00670 |
| 1-heptadecanoyl-2-hydroxy-sn-glycero-3-phosphocholine | 510.35623 | C_25_H_52_NO_7_P | 0.01130 | 0.06206 | 2.01786 | NA |
| Clomipramine | 315.16013 | C_6_H_11_NO_3_S | 0.01297 | 0.04922 | 1.55992 | C06918 |
| Propionylcarnitine | 218.13936 | C_10_H_19_NO_4_ | 0.01361 | 0.22766 | 1.63867 | C03017 |
| 13a-Hydroxylupanin | 265.19141 | C_15_H_24_N_2_O_2_ | 0.01526 | 1.56470 | 1.20359 | NA |
| NCGC00385811-6-[3-[(3,4-dimethoxyphenyl)methyl]-4-methoxy-2-(methoxymethyl)butyl]-4-methoxy-1,3-benzodioxole | 415.21201 | C_24_H_32_O_7_ | 0.01604 | 6.12070 | 1.63590 | NA |
| phenylacetylglutamine | 265.11777 | C_13_H_16_N_2_O_4_ | 0.01719 | 0.13587 | 1.66913 | C04148 |
| N4-AcetylSulfameth | 296.06952 | C_12_H_13_N_3_O_4_S | 0.02074 | 3.53890 | 1.65147 | C13061 |
| Sulfamethoxazole | 254.05939 | C_10_H_11_N_3_O_3_S | 0.02500 | 2.69330 | 1.67394 | C07315 |
| Trimethoprim | 291.14417 | C_14_H_18_N_4_O_3_ | 0.02510 | 1.67820 | 1.89264 | C01965 |
| Prolylhydroxyproline | 229.11839 | C_10_H_16_N_2_O_4_ | 0.03232 | 0.10647 | 1.07477 | NA |
| Heptadecanoic acid | 271.27484 | C_17_H_34_O_2_ | 0.03696 | 2.00420 | 1.66516 | NA |
| Diaveridine | 261.13074 | C_13_H_16_N_4_O_2_ | 0.03792 | 0.08594 | 1.55843 | NA |
| 1-heptadecanoyl-2-hydroxy-sn-glycero-3-phosphocholine | 510.35593 | C_25_H_52_NO_7_P | 0.03797 | 0.07164 | 1.79894 | NA |
| Pesticide3_Pirimicarb_C11H18N4O2_2-(Dimethylamino)-5,6-dimethyl-4-pyrimidinyl dimethylcarbamate | 261.13046 | C_11_H_18_N_4_O_2_ | 0.04495 | 0.15680 | 1.38496 | NA |
| L-kynurenine | 209.08992 | C_10_H_12_N_2_O_3_ | 0.04669 | 0.43880 | 1.33426 | C00328 |
| NCGC00385811-01!6-[3-[(3,4-dimethoxyphenyl)methyl]-4-methoxy-2-(methoxymethyl)butyl]-4-methoxy-1,3-benzodioxole | 415.21185 | C_24_H_32_O_7_ | 0.04773 | 3.40760 | 1.30344 | NA |

**Table S3** Differential metabolites between plasma of patients in remission and non-remission

| **Name** | **m/z** | **Formula** | ***P-value*** | **FC** | **VIP** | **KEGG ID** |
| --- | --- | --- | --- | --- | --- | --- |
| LPC(16:0) | 496.34100 | C_24_H_50_NO_7_P | 0.00142 | 1.81090 | 1.86691 | C04230 |
| Tribenzylamine | 288.17532 | C_21_H_21_N | 0.00255 | 0.39932 | 1.48250 | NA |
| Tetradecanoyl-L-Carnitine | 372.31149 | C_21_H_41_NO_4_ | 0.00532 | 2.23965 | 1.65269 | NA |
| LPC(16:0) | 496.34128 | C_24_H_50_NO_7_P | 0.00701 | 1.74350 | 1.68081 | C04230 |
| 4H-cyclopenta[def]phenanthrene-4-one | 205.06879 | C_15_H_8_O | 0.00757 | 0.00574 | 1.49875 | NA |
| LPC(16:0) | 496.34036 | C_24_H_50_NO_7_P | 0.00985 | 1.66857 | 1.70011 | C04230 |
| N,N-Dimethylarginine | 203.15025 | C_8_H_18_N_4_O_2_ | 0.01029 | 0.29213 | 1.49601 | C03626 |
| LPC(16:0) | 496.34094 | C_24_H_50_NO_7_P | 0.01237 | 1.78499 | 1.62162 | C04230 |
| 1-Stearoyl-sn-glycero-3-phosphocholine | 524.37195 | C_26_H_54_NO_7_P | 0.01254 | 1.64342 | 1.44693 | NA |
| LPC(16:0) | 496.33826 | C_24_H_50_NO_7_P | 0.01289 | 1.49256 | 1.61204 | C04230 |
| Oleoylcarnitine | 426.35828 | C_25_H_47_NO_4_ | 0.01297 | 3.16553 | 1.42370 | NA |
| Palmitoylcarnitine | 400.34210 | C_23_H_45_NO_4_ | 0.01690 | 2.32067 | 1.43905 | C02990 |
| Pseudouridine | 245.07037 | C_9_H_12_N_2_O_6_ | 0.02677 | 0.16459 | 1.48328 | C02067 |
| 13a-Hydroxylupanin | 265.19150 | C_15_H_24_N_2_O_2_ | 0.03093 | 2.51356 | 1.37757 | NA |
| 1-heptadecanoyl-2-hydroxy-sn-glycero-3-phosphocholine | 510.35614 | C_25_H_52_NO_7_P | 0.03371 | 1.62058 | 1.57802 | NA |
| Melibiose | 365.10464 | C_12_H_22_O_11_ | 0.03628 | 0.42402 | 1.20255 | C05400 |
| 1-pentadecanoyl-2-hydroxy-sn-glycero-3-phosphocholine | 482.32465 | C_23_H_48_NO_7_P | 0.03804 | 1.88729 | 1.52977 | NA |
| Trimethoprim | 291.14417 | C_14_H_18_N_4_O_3_ | 0.03880 | 0.00583 | 1.22611 | C01965 |
| Cilastatin | 200.04068 | C_6_H_11_NO_3_S | 0.04466 | 0.08965 | 1.34090 | C01675 |
| Oxymetholone | 331.21765 | C_21_H_32_O_3_ | 0.04518 | 0.78178 | 1.10856 | NA |
| Tubocurarine | 609.29352 | C_37_H_40_N_2_O_6_ | 0.04692 | 0.08012 | 1.53626 | NA |
