# Supplementary material for: CAR-T therapy alters synthesis of platelet-activating factor in multiple myeloma patients
Source: J Hematol Oncol. 2021 Jun 9;14:90. doi: 10.1186/s13045-021-01101-6 (PMC8191024; doi:10.1186/s13045-021-01101-6)
Supplement: Supplementary file 3 — Additional file 3. Lipidomics. [file 13045_2021_1101_MOESM3_ESM.docx]

**Additional file 3:** Lipidomics

Sample preparation

There were 12 plasma samples from patients in remission and 5 plasma samples from patients in non-remission at 30 days after CAR T cell infusion. An aliquot of 20 μL frozen plasma sample was mixed with 225 μL of ice-cold methanol (Merck, USA) containing mixture of odd chain and deuterated lipids internal standards (LPE(17:1), SM(17:0), PE(17:0/17:0), purchased from Avanti Polar Lipids Company, USA). The mixture was vortexed for 10 s. Then, adding 750 μL of methyl tert-butyl ether (MTBE, Merck, USA) to the mixture. The mixture was vortexed for 10 s and shaken at 4°C for 10 min. After addition of 188 μL of ultrapure water, the samples were vortexed and centrifuged at 18,000 rpm at 4°C for 2min. Finally, an aliquot of 350 μL upper phase was transferred to fresh tube and dried in SpeedVac sample concentrator at 45°C for 2 h. The residue was reconstituted in 110 μL of a mixture of methanol/toluene (9:1, vol/vol) (Merck, USA) for HPLC-MS analysis.

HPLC-MS analysis

UltiMate 3000 HPLC system (Dionex, USA) coupled online via ESI with an Q Exactive™ HF MS instrument (Thermo Fisher Scientific, USA) was applied for untargeted lipidomic analysis. For the lipids of plasma, an aliquot of 2 µL sample solution was injected onto a reversed phase Waters Acquity HPLC CSH C18 (100 mm×2.1 mm, 1.7 μm) maintained at 65°C. The auto-sampler was conditioned at 4°C. For analysis carried out in the positive ESI mode, the mobile phase was composed of A (40% acetonitrile in water containing 10 mM ammonium formate (ROE, USA) and 0.1% formic acid) and B (a mixture of isopropanol (ROE, USA)/acetonitrile (9:1, vol/vol) including 10 mM ammonium formate (ROE, USA) and 0.1% formic acid). The gradient profile was as follows: 15-30%B at 0-4 min, 30-48%B at 4-5 min, 48%-82%B at 5-22 min, 82%-99%B at 22-23 min, 99%B at 23-24 min, 99%-15%B at 24-24.2 min, and 15%B at 24.2-30 min. The flow rate was 0.3 mL/min.

All MS experiments were performed in positive ion mode using a heated electrospray ionization source. The source and ion transfer parameters applied were as follows: spray voltage 3.5 kV, the sheath gas, aux gas, the capillary temperature and the heater temperature were maintained at 45 arb, 10 arb, 300°C and 306°C, respectively. The S-Lens RF level was set at 50. The Orbitrap mass analyzer was operated at a resolving power of 60,000 in full-scan mode (scan range: 215-1800 m/z).

Data processing and statistics

Lipid annotation was performed by the query of the exact mass of the detected features against LipidSearch (Version 4.1; ThermoFisher Scientific, USA). After lipid annotation, the raw data files were converted and automated peak picking, integration, RT adjustment by the aforementioned database and alignment were conducted with MS-DIAL (version 1.92). In this study, only a lipid feature defined as an accurate mass to charge ratios (m/z)-RT pair could be aligned for an identical lipid. The resulting output data table of high-quality time-aligned examined lipids, with their peak height intensity was normalized by median peak intensity of sample. Then, the data set of normalized peak height intensity, metabolites names and sample numbers were analyzed by SIMCA 14.1 software. SIMCA 14.1 performed a multivariate statistical analysis of the PCA and OPLS-DA and permutations (Fig.S3). The lipids with *P* value < 0.05 and VIP of >1.0 were considered as statistically significant lipids. Finally, the significant lipids with FC >1.5 or <0.67 were deemed as potential biomarkers. Volcano plot was carried out on the website visualization tools of MetaboAnalyst 5.0. (Table S4).


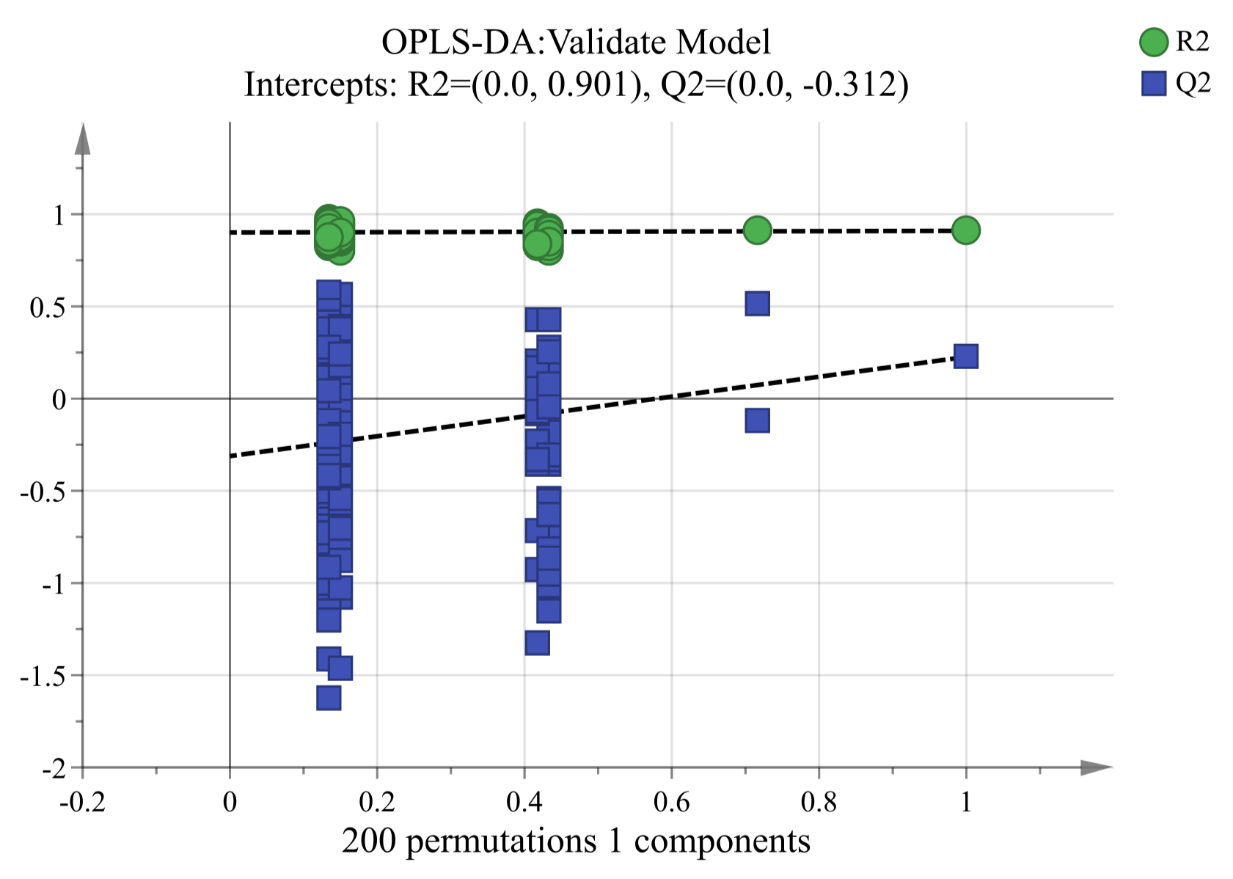


**Fig. S3** Validation of OPLS-DA model in comparative efficacy group by 200 permutation tests. R2 = 0.901, Q2 = -0.312.

**Table S4** Differential lipids between plasma of patients in remission and non-remission

| **Name** | **m/z** | **Formula** | ***P-value*** | **FC** | **VIP** |
| --- | --- | --- | --- | --- | --- |
| PC(36:4) | 804.54834 | C_44_H_80_NO_8_P | 0.00019 | 2.14430 | 1.96594 |
| PC(36:4) | 782.56744 | C_44_H_80_NO_8_P | 0.00036 | 1.87385 | 1.88026 |
| TG(54:3)\|TG(18:1_18:1_18:1) | 902.81451 | C_57_H_104_O_6_ | 0.00073 | 0.63550 | 1.75056 |
| TG(54:3)\|TG(18:0_18:1_18:2) | 907.77551 | C_57_H_104_O_6_ | 0.00174 | 0.65585 | 1.99237 |
| PC(35:4) | 768.55688 | C_43_H_78_NO_8_P | 0.00253 | 2.10194 | 1.81024 |
| TG(54:1)\|TG(18:0_18:0_18:1) | 911.80981 | C_57_H_108_O_6_ | 0.00272 | 0.50905 | 1.90211 |
| PC(38:6) | 828.55286 | C_46_H_80_NO_8_P | 0.00273 | 1.99067 | 1.74872 |
| TG(52:1)\|TG(16:0_18:0_18:1) | 883.77704 | C_55_H_104_O_6_ | 0.00498 | 0.57842 | 1.81080 |
| PE(36:2) | 766.53137 | C_41_H_78_NO_8_P | 0.00524 | 0.59576 | 1.87641 |
| TG(54:1)\|TG(16:0_20:0_18:1) | 906.85559 | C_57_H_108_O_6_ | 0.00570 | 0.52771 | 1.78438 |
| LPC(17:0/0:0) | 510.35446 | C_25_H_52_NO_7_P | 0.00608 | 0.57530 | 1.40278 |
| TG(54:1)\|TG(16:0_18:0_20:1) | 906.84375 | C_57_H_108_O_6_ | 0.00687 | 0.53067 | 1.76369 |
| PC(38:5) | 830.56970 | C_46_H_82_NO_8_P | 0.00744 | 1.59701 | 1.67351 |
| TG(56:4)\|TG(18:0_18:0_20:4) | 928.83490 | C_59_H_106_O_6_ | 0.00927 | 0.56025 | 1.66729 |
| PC(40:7) | 854.57483 | C_48_H_82_NO_8_P | 0.00936 | 1.67909 | 1.58647 |
| TG(54:2)\|TG(18:0_18:1_18:1) | 909.78912 | C_57_H_106_O_6_ | 0.01295 | 0.62326 | 1.79619 |
| PC(38:6) | 806.56445 | C_46_H_80_NO_8_P | 0.01494 | 1.98492 | 1.40661 |
| SM(42:5;2O)\|SM(24:3;2O/18:2) | 807.63177 | C_47_H_87_N_2_O_6_P | 0.01502 | 1.56279 | 1.16916 |
| PC(O-38:4) | 796.62085 | C_46_H_86_NO_7_P | 0.01509 | 0.64362 | 1.38876 |
| PC(O-46:9) | 898.66327 | C_54_H_92_NO_7_P | 0.01598 | 3.52704 | 1.38918 |
| TG(60:4)\|TG(18:1_24:1_18:2) | 984.90100 | C_63_H_114_O_6_ | 0.01638 | 0.48421 | 1.68978 |
| TG(46:2)\|TG(14:0_16:1_16:1) | 797.66748 | C_49_H_90_O_6_ | 0.01846 | 0.56355 | 1.55946 |
| PC(38:6) | 806.57550 | C_46_H_80_NO_8_P | 0.01862 | 2.02145 | 1.40963 |
| PC(38:6) | 828.55316 | C_46_H_80_NO_8_P | 0.01916 | 1.54544 | 1.46648 |
| SM(42:4;2O)\|SM(20:2;2O/22:2) | 809.64764 | C_47_H_89_N_2_O_6_P | 0.02234 | 1.50670 | 1.31211 |
| TG(60:4)\|TG(18:1_24:1_18:2) | 984.90198 | C_63_H_114_O_6_ | 0.02239 | 0.47457 | 1.62877 |
| TG(55:3)\|TG(18:1_18:1_19:1) | 916.81903 | C_58_H_106_O_6_ | 0.02401 | 0.60523 | 1.69153 |
| TG(52:1)\|TG(16:0_18:0_18:1) | 878.81555 | C_55_H_104_O_6_ | 0.02565 | 0.58234 | 1.60342 |
| SM(42:1;2O)\|SM(18:1;2O/24:0) | 815.70038 | C_47_H_95_N_2_O_6_P | 0.02606 | 1.53880 | 1.42826 |
| PC(38:4) | 810.59882 | C_46_H_84_NO_8_P | 0.02940 | 1.59405 | 1.42092 |
| TG(58:2)\|TG(16:0_18:1_24:1) | 960.90198 | C_61_H_114_O_6_ | 0.03546 | 0.65576 | 1.52980 |
| PC(37:5) | 794.57104 | C_45_H_80_NO_8_P | 0.03607 | 1.57008 | 1.53639 |
| LPC(O-24:0) | 594.48474 | C_32_H_68_NO_6_P | 0.03637 | 2.72463 | 1.16617 |
| LPC(20:4) | 566.32336 | C_28_H_50_NO_7_P | 0.03656 | 1.60117 | 1.30817 |
| PC(O-42:4) | 852.68121 | C_50_H_94_NO_7_P | 0.03686 | 1.99490 | 1.34140 |
| LPC(20:4/0:0) | 544.33777 | C_28_H_50_NO_7_P | 0.04162 | 1.57376 | 1.28808 |
| CE(18:2) | 666.62189 | C_45_H_76_O_2_ | 0.04233 | 1.54481 | 1.51810 |
| CAR(26:0) | 540.49872 | C_33_H_66_NO_4_ | 0.04433 | 3.05955 | 1.27285 |
| TG(60:3)\|TG(24:0_18:1_18:2) | 986.91217 | C_63_H_116_O_6_ | 0.04461 | 0.64096 | 1.51107 |
| LPC(16:1/0:0) | 494.32516 | C_24_H_48_NO_7_P | 0.04482 | 1.55125 | 1.32919 |
| PE(P-38:5)\|PE(P-18:1_20:4) | 750.54370 | C_43_H_76_NO_7_P | 0.04493 | 1.75713 | 1.37642 |
| SM(41:3;2O) | 813.63995 | C_46_H_89_N_2_O_7_P | 0.04529 | 1.78453 | 1.07518 |
| SM(40:7;2O) | 791.57465 | C_45_H_79_N_2_O_7_P | 0.04557 | 1.63650 | 1.31613 |
| CE(20:4) | 690.61627 | C_47_H_76_O_2_ | 0.04603 | 3.29186 | 1.25606 |
