# Supplementary material for: CAR-T therapy alters synthesis of platelet-activating factor in multiple myeloma patients
Source: J Hematol Oncol. 2021 Jun 9;14:90. doi: 10.1186/s13045-021-01101-6 (PMC8191024; doi:10.1186/s13045-021-01101-6)
Supplement: Supplementary file 4 — Additional file 4. GEP analysis of other 3 members in LPCAT family including LPCAT2, LPCAT3 and LPCAT4. [file 13045_2021_1101_MOESM4_ESM.docx]

**Additional file 4:** GEP analysis of other 3 members in LPCAT family including LPCAT2, LPCAT3 and LPCAT4.


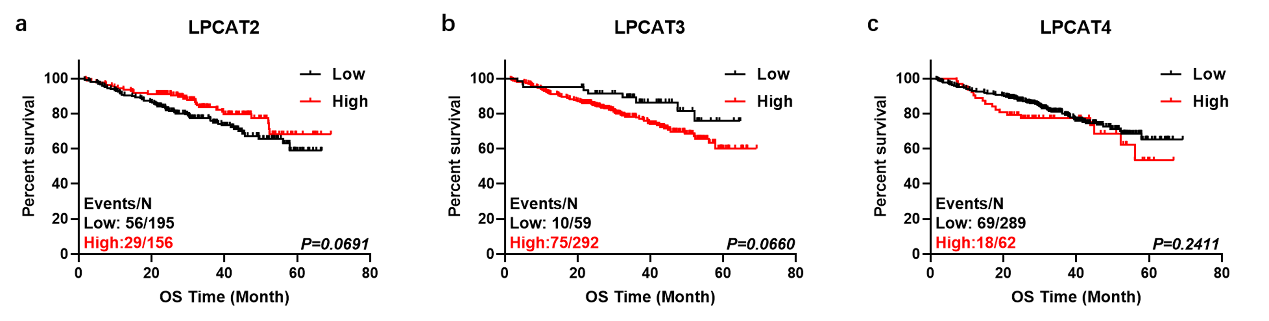


**Fig. S4** The expression of LPCAT2, LPCAT3 and LPCAT4 in MM patients was not significantly associated with poor OS in TT2 cohort.
